# Recovery of brain DHA-containing phosphatidylserine and ethanolamine plasmalogen after dietary DHA-enriched phosphatidylcholine and phosphatidylserine in SAMP8 mice fed with high-fat diet

**DOI:** 10.1186/s12944-020-01253-3

**Published:** 2020-05-25

**Authors:** Ying-Cai Zhao, Miao-Miao Zhou, Ling-Yu Zhang, Pei-Xu Cong, Jie Xu, Chang-Hu Xue, Teruyoshi Yanagita, Naiqiu Chi, Tian-Tian Zhang, Feng-Hai Liu, Yu-Ming Wang

**Affiliations:** 1 College of Food Science and Engineering, Ocean University of China, Qingdao, 266003 Shandong China; 2 Centre for Nutrition and Food Sciences, Queensland Alliance for Agriculture and Food Innovation, The University of Queensland, Brisbane, QLD 4072 Australia; 3 Laboratory for Marine Drugs and Bioproducts, Pilot National Laboratory for Marine Science and Technology (Qingdao), Qingdao, 266237 Shandong Province People’s Republic of China; 4 Laboratory of Nutrition Biochemistry, Department of Applied Biochemistry and Food Science, Saga University, Saga, 840-8502 Japan; 5 Qingdao Silver Century Health Industry Group Co., Ltd., Qingdao, 266110 Shandong Province People’s Republic of China; 6 Department of Clinical Laboratory, Qingdao Municipal Hospital (Group), Qingdao, 266011 Shandong Province People’s Republic of China

**Keywords:** Alzheimer’s disease, Lipid profile, DHA, Phosphatidylserine, Phosphatidylcholine

## Abstract

**Background:**

Glycerophospholipids were the main components of cerebral cortex lipids, and there was a close association between lipid homeostasis and human health. It has been reported that dietary DHA-enriched phosphatidylcholine (DHA-PC) and phosphatidylserine (DHA-PS) could improve brain function. However, it was unclear that whether supplementation of DHA-PC and DHA-PS could change lipid profiles in the brain of dementia animals.

**Methods:**

SAMP8 mice was fed with different diet patterns for 2 months, including high-fat diet and low-fat diet. After intervention with DHA-PC and DHA-PS for another 2 months, the lipid profile in cerebral cortex was determined by lipidomics in dementia mice.

**Results:**

High-fat diet could significantly decrease the levels of DHA-containing PS/pPE, DPA-containing PS, and AA-containing PE, which might exhibit the potential of lipid biomarkers for the prevention and diagnosis of AD. Notably, DHA-PC and DHA-PS remarkably recovered the lipid homeostasis in dementia mice. These might provide a potential novel therapy strategy and direction of dietary intervention for patients with cognitive decline.

**Conclusions:**

DHA-PC and DHA-PS could recover the content of brain DHA-containing PS and pPE in SAMP8 mice fed with high-fat diet.

**Abstract graphical:**

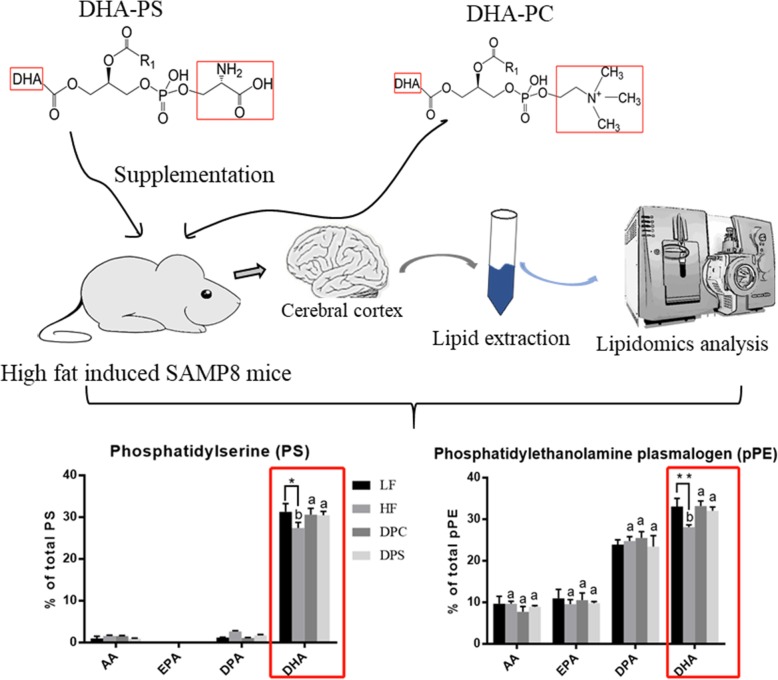

## Introduction

Alzheimer’s disease (AD) is a progressive multifarious neurodegenerative disorder [1]. The clinical manifestations of AD are characterized with memory impairment, aphasia, agnosia, visual spatial skills impairment, executive dysfunction, personality and behavioral changes, etc. [2]. Long-term care insurance for the dementia patients will be a serious social burden following the aging society. Alzheimer’s patients had significant lipid changes in the brain tissue along with the pathological changes. Dietary habits could affect the occurrence and development of AD, and the effect of lipids on brain pathology of AD patients has been controversial. Several studies have suggested that high-fat diet could increase the risk of AD, while others have found that specific structural lipids might have a protective effect on cognitive function via antagonizing the effects of western diet [3, 4]. The different effects of high-fat diet might be attributed to the saturation of fatty acids and the molecular types of lipids.

DHA is the most abundant omega-3 fatty acid in the brain [5]. As a major component for the growth and maintenance of nervous system, docosahexaenoic acid (DHA) can cross the blood-brain barrier through the Mfsd2a to provide the raw material for the formation of neuronal cell membrane [6]. Phosphatidylcholine (PC), transported by phosphatidylcholine transporter between membranes, is the main component of biofilm. It is sufficient to activate the endogenous ligand for the nuclear receptor peroxisome proliferator-activated receptors (PPAR-α) in hepatocytes [7]. Phosphatidylserine (PS) is the major acidic phospholipid class that accounts for 13–15% of the phospholipids in the human cerebral cortex, and it is localized exclusively in the inner leaflet of the plasma membrane in neural tissues [8]. As part of protein docking site in signal pathways, PS can interact with Akt, Raf and protein kinase C to ensure the growth and differentiation of nerve cells [9]. It has been reported that DHA enriched phospholipids could suppress the activation of microglia and astrocytes, and ameliorate Aβ-induced neuroinflammation via inhibiting the proinflammatory factors such as IL1β and TNFα [10]. Our previous study showed that DHA-PS (DHA enriched phosphatidylserine) has more significant effect than DHA-PC (DHA enriched phosphatidylcholine) on alleviating Aβ-induced cognitive deficiency [11]. DHA was mainly integrated with PS in nervous system, total PS level in brain tissue would increase after supplementation with DHA [12]. In an open-label extension study, daily intake of 100 mg DHA-PS could improve memory and maintain cognitive level in dementia patients [13]. However, it was unclear that whether supplementation of DHA-PC and DHA-PS could change lipid profiles in the brain of dementia animals.

Lipidomics could systematically identify the key lipid biomarkers in pathological process by determining lipid profiles, and may ultimately demonstrate the functional properties in different physiological status. Various studies have revealed the momentous changes of lipids in brain tissue and serum of dementia patients during the pathological process of AD using lipidomics [14]. SAMP8 is a naturally occurring mouse line that displays a phenotype of accelerated aging and is consistent with the pathological characteristics of AD. The objective of this study was to elucidate the effects of dietary DHA-PC and DHA-PS on the changes of AD brain lipid profile, and lipidomic analysis was carried out to evaluate the lipid composition in cerebral cortex of cognitive deficiency model SAMP 8 mice fed with high-fat diet.

## Materials and method

### Preparation of DHA-PC and DHA-PS

Total lipid was exacted from squid (*Stenoteuthis. oualaniensis*) roe according to the method of Folch [15]. DHA-PC was then purified from total lipid via silica-gel column chromatography with the successively multiple elution of chloroform, acetone and methanol. DHA-PS was synthesized from DHA-PC by phospholipase D as the method described by Wang et al. [16]. The purity of DHA-PC and DHA-PS was confirmed to be 94.7 and 95.1% by HPLC-ELSD [17].

### Fatty acid analysis

The fatty acid compositions of DHA-PC and DHA-PS were analyzed by gas chromatography. Extracted lipid was dissolved and methylated with 2 mol/L hydrochloric acid in methanol for 1 h before injecting into gas chromatography Agilent 7820A (Agilent Technologies Inc.) with a flame ionization detector. Nitrogen was used as carrier gas. The analysis was carried out with a capillary column Supelcowax (30 m × 0.32 mm I.D and 0.25 μm film thickness; Sigma-Aldrich Inc). The temperature of injector and detector was 230 °C and 250 °C, respectively. The temperature of oven started from 170 to 230 °C at the rate of 3 °C/min and retained at 230 °C for 20 min. The relative content of each fatty acid was shown in Table 1.

**Table 1:** Main fatty acid composition of DHA-PC and DHA-PS

fatty acid composition (%)	DHA-PC	DHA-PS
Palmitic acid (C16:0)	28.08 ± 0.26	28.16 ± 0.17
Palmitoleic acid (C16:1)	0.54 ± 0.01	0.67 ± 0.02
Stearic acid (C18:0)	15.56 ± 0.11	15.38 ± 0.14
Arachidonic acid	1.54 ± 0.07	1.63 ± 0.08
Oleic acid (C18:1)	1.37 ± 0.03	1.64 ± 0.05
EPA (C20:5)	11.62 ± 0.09	12.09 ± 0.11
DHA (C22:6)	35.28 ± 0.35	34.48 ± 0.47
other fatty acid	7.55 ± 0.21	7.58 ± 0.19

### Animals and diets

All male SAMP8 mice (20 ± 2 g, 8 months) were housed at SPF animal room under the temperature of 20 ± 2 °C and the humidity of 60% with a 12/12 h light/dark cycle (light starting at 8 a.m.). At the adaptation period, 8 mice were fed with low fat diet and other 24 mice were treated with high-fat diet. At the age of 10 month, the SAMP8 mice were divided into 4 groups (8 animals per group): low fat group (LF) fed with standard diet (AIN-93 M), high-fat group (HF) fed with high-fat diet, DPC group fed with high-fat diet containing 1% (w/w) DHA-PC and DPS group fed with high-fat diet containing 1% (w/w) DHA-PS. The mice were continuously treated with corresponding diets for another 2 months. The ingredients of each diet were summarized in Table 2 and all the four groups got the same treatment during animal feeding period. The study protocols were approved by the ethical committee of experimental animal care at Ocean University of China (Qingdao, China). After 2 months of intervention, animals were sacrificed and the cortex were quickly separated from brain tissue on ice, then were frozen in liquid nitrogen and stored at − 80 °C.

**Table 2:** Composition of experimental diets (g/kg diet)

Ingredients g/kg	LF	HF	DHA-PC	DHA-PS
Casein	140.0	140.0	140.0	140.0
Corn starch	617.7	430.7	430.7	430.7
Sucrose	100.0	100.0	100.0	100.0
Corn oil	23.9	46.0	46.0	46.0
Lard	19.1	184.0	174.0	174.0
Mineral mix^a^	35.0	35.0	35.0	35.0
Vitamin mix^b^	10.0	10.0	10.0	10.0
Cellulose	50.0	50.0	50.0	50.0
L-cystine	2.5	2.5	2.5	2.5
Choline bitartrate	1.8	1.8	1.8	1.8
t-butylhydroquinone	0.008	0.008	0.008	0.008
DHA-PC	–	–	10.0	–
DHA-PS	–	–	–	10.0

Note:“-”, not detected

^a^ AIN-93 M mineral mix

^b^ AIN-93 M vitamin mix

### Cerebral cortex lipid extraction

The cerebral cortex was crushed in phosphate buffer using tissue homogenizer. The total lipid was extracted from cerebral cortex using a modified Bligh/Dyer procedure [18]. Briefly, the tissue homogenate was extracted with 20-fold volume of chloroform: methanol 2:1 (v/v) and incubated for 1 h in a thermomixer. The mixture was then centrifuged at 10000 g, 4 °C for 5 min. Deionized H_2_O was added to the obtained supernatant before vortex. After centrifugation, the lower organic phase were vacuumed to remove the solvent. The obtained lipids were then dissolved in water/acetonitrile/isopropanol 1:1:2 (v/v/v) for further analysis.

### Lipidomic analysis

The polar lipids were separated by NP-HPLC using Phenomenex Luna 3 μm micron silica column (150 × 2.0 mm; Phenomenex Inc.) treated with mobile phase A chloroform: methanol: ammonia water (89.5:10:0.5) and mobile phase B chloroform: ethanol: ammonia water: water (55:39:0.5:5.5) at a flow rate of 0.25 mL/min. Gradient elution was performed with 5% B for 5 min, a step to 40% B until 12 min and maintained for 4 min, a linear decrease to 30% B and maintained for 15 min, and re-equilibration from 33 min to 38 min with 5% B. Mass spectrometry multiple reaction monitoring (MRM) mode was established for comparative analysis of various polar lipids.

All experiments were carried out by Exion UPLC-QTRAP 6500 PLUS (Sciex Inc.) liquid chromatography-mass spectrometry with the analysis carried out in the electrospray ionization (ESI) mode. The conditions were as follows: curtain gas = 20 psi, ion spray voltage = 5500 V, turbo spray source temperature = 400 °C, ion source gas 1 = 35 psi, ion source gas 2 = 35 psi [19].

### Statistical analysis

Results were presented as mean and standard deviation (Mean ± SD) in tables and figures. The comparison between LF and HF groups was carried out by student’s t-test *P < 0.05, **P < 0.01. Differences among three groups HF, DPC and DPS were tested by one-way ANOVA (Turkey’s test) and different letters (a, b, c) indicated different significance at P < 0.05.

## Results

### Alterations of glycerophospholipids

**Fig. 1:**
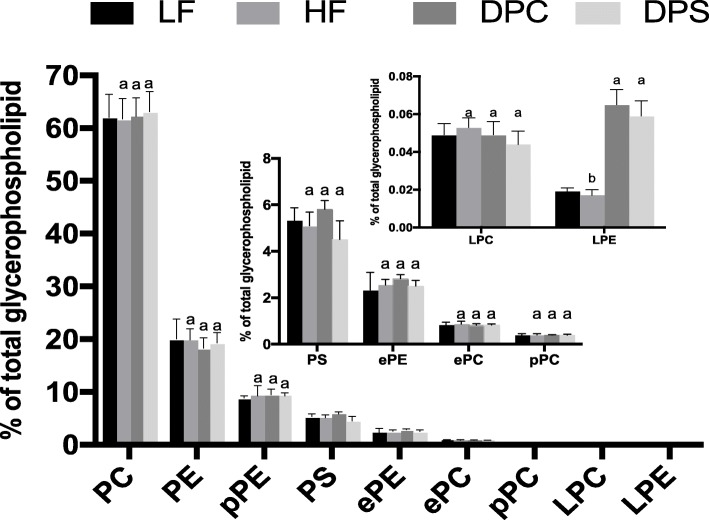
The alteration of glycerophospholipid classes in cerebral cortex of SAMP8 mice in four groups. Glycerophospholipid composition was measured following two 2-month dietary intervention. Results were presented as mean and standard deviation (*n* = 8). The comparison of LF and HF group was tested by unpaired two-tailed student’s *t-*test. Different letters among HF, DPC and DPS groups represented significant difference at *P* < 0.05 determined by one-way ANOVA (Tukey’s test)

Glycerophospholipids, such as phosphatidylcholine, phosphatidylethanolamine (PE) and phosphatidylinositol, is one of the most abundant lipid classes in cerebral cortex. After 8 weeks of DHA-PC and DHA-PS intervention, the alteration of glycerophospholipid molecular species in the cerebral cortex was shown in Fig. 1. There were only small changes with no significance in the levels of the main glycerophospholipid classes. PC and PE are the primary constituents in the cerebral cortex and account for more than 60% of the total glycerophospholipid as represented. The result indicated that the contents of PC and PE in cerebral cortex were not easily affected by exogenous phospholipids intake (Fig. 1**)**. PS, a brain-specific nutrition, is abundant in brain tissue and is essential in terms of memory function, which account for 10–20% of total phospholipids in the brain. Nevertheless, dietary DHA-PS did not have much impact on the changes of molecular species. DHA-PC supplementation could slightly improve the PS instead of PC content but with no significance in cerebral cortex compared with DHA-PS (Fig. 1**)**. However, the PS levels were did not changed among the four groups.

Despite relatively low abundance, lyso-phosphatidylethanolamine (LPE) exhibited the most obvious change in lipid classes (Fig. 1**)**. Supplementation of DHA-PC and DHA-PS significantly raised the content of LPE (*P* < 0.05) in comparison with LF and HF groups, which might be attributed to the specialty of its direct transfer into brain tissue [20]. After digestion and absorption, glycerophospholipids were hydrolyzed to form lysophospholipid, which could be transported to various tissues and organs in the form of chylomicron in the circulatory system. Lysophospholipid could enter the brain through the blood-brain barrier and be reused to synthesize new lipids. This property might be the reason for changes of lysophospholipid content after intake of DHA enriched phospholipids.

### The fatty acid composition of each glycerophospholipid

**Fig. 2:**
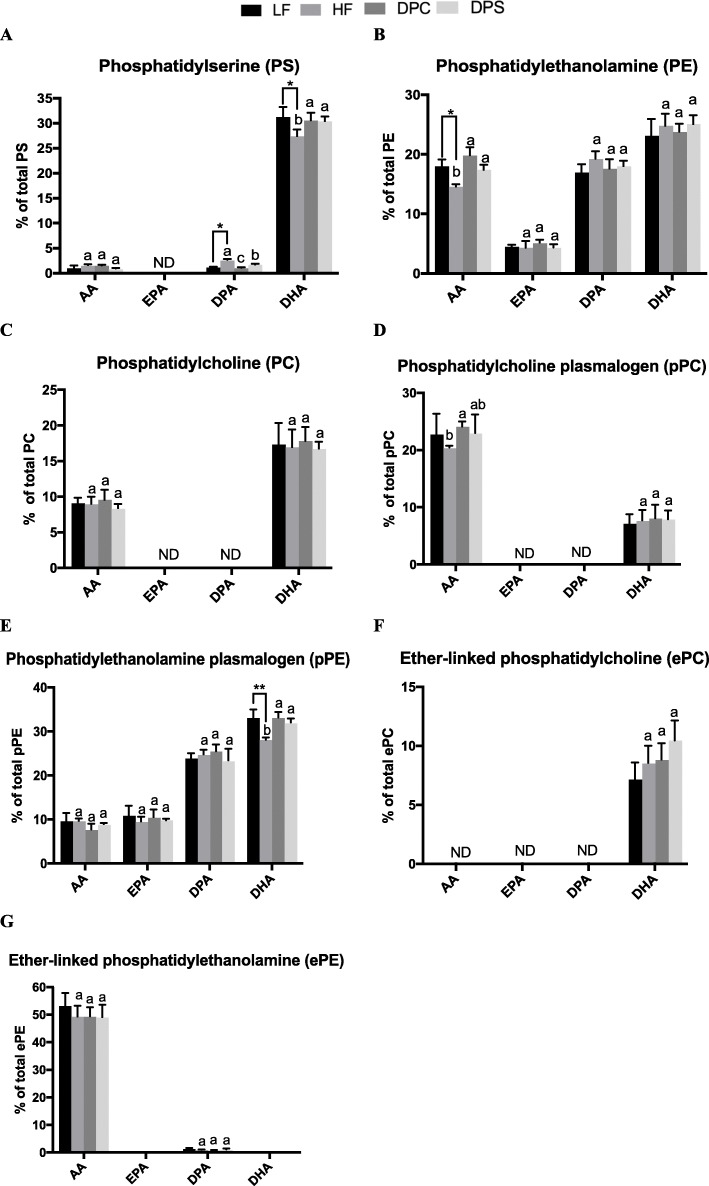
The relative abundance of polyunsaturated fatty acid (PUFA) including AA, EPA, DPA and DHA attached glycerophospholipid following different dietary interventions within 2 months. **a** The relative percentage of each PUFA attached phosphatidylserine (PS) occupied in total PS of cerebral cortex. **b** The relative percentage of each PUFA attached phosphatidylethanolamine (PE) occupied in total PE of cerebral cortex. **c** The relative percentage of each PUFA attached phosphatidylcholine (PC) occupied in total PC of cerebral cortex. **d** The relative percentage of each PUFA attached phosphatidylcholine plasmalogen (pPC) occupied in total pPC of cerebral cortex. **e** The relative percentage of each PUFA attached phosphatidylethanolamine plasmalogen (pPE) occupied in total pPE of cerebral cortex. **f** The relative percentage of each PUFA attached ether-linked phosphatidylcholine (ePC) occupied in total pPE of cerebral cortex. **g** The relative percentage of each PUFA attached ether-linked phosphatidylethanolamine (ePE) occupied in total ePE of cerebral cortex. ND meant that substance were not detected. Results were presented as mean and standard deviation (n = 8). The comparison of LF and HF group was tested by unpaired two-tailed student’s *t*-test *P<0.05;**P<0.01; Different letters among HF, DPC and DPS groups represented significant difference at *P* < 0.05 determined by ANOVA (Tukey’s test)

As for PS molecular species, 18:0/18:1, 18:0/20:3, 18:0/22:6 and 20:1/18:2 are the main fatty acid species as shown in Table 3. Consistent with the previous study, 18:0/22:6 has the highest abundance with the content of 28% in PS, and DHA is the predominated polyunsaturated fatty acid (Fig. 2) [21]. Although total PS level did not appreciably change after intervention with different diets for 2 months (Fig. 1), DHA-PC/PS group could significantly change the fatty acid composition of PS, especially the polyunsaturated fatty acids composition (Table 3**)**. High-fat diet reduced the relative content of 18:0/22:6-PS. Interestingly, the level of 18:0/22:6-PS could be restored to that of LF group after supplementation with DHA enriched phospholipids.

**Table 3:** The lipid profile of PS in cerebral cortex after dietary intake of DHA enriched phospholipids

Fatty Acid (%)	LF	HF	DPC	DPS
	Mean ± SD	Mean ± SD	Mean ± SD	Mean ± SD
**18:0/18:1**	**8.02 ± 0.85**	**8.67 ± 0.61**^**a**^	**6.13 ± 0.11**^**b**^	**8.66 ± 1.21**^**a**^
18:0/20:1	1.90 ± 0.64	2.44 ± 0.10 ^**a**^	1.49 ± 0.17 ^**b**^	2.11 ± 0.13 ^**ab**^
**18:0/20:3**	**26.56 ± 1.66**	**26.03 ± 1.51**	**27.34 ± 1.60**	**25.77 ± 3.42**
18:0/20:4	0.97 ± 0.56	1.59 ± 0.11	1.57 ± 0.12	0.95 ± 0.10
18:0/22:4	2.06 ± 0.31	2.51 ± 0.28 ^**a**^	1.53 ± 0.10 ^**b**^	2.10 ± 0.30 ^**ab**^
18:0/22:5	1.19 ± 0.10	2.66 ± 0.15 ^**a^	1.09 ± 0.12 ^**c**^	1.73 ± 0.19 ^**b**^
**18:0/22:6**	**28.93 ± 1.86**	24.98 ± 0.86^*b^	**28.52 ± 1.25**^**a**^	**28.25 ± 1.35**^**a**^
18:1/18:1	1.49 ± 0.14	1.91 ± 0.25	1.55 ± 0.16	1.67 ± 0.08
18:1/20:3	0.88 ± 0.29	0.77 ± 0.13	0.53 ± 0.07	0.80 ± 0.11
18:1/22:6	0.88 ± 0.23	0.77 ± 0.15	0.49 ± 0.11	0.81 ± 0.12
**20:1/18:2**	**25.65 ± 2.68**	**26.02 ± 1.91**	**28.54 ± 1.90**	**25.77 ± 1.22**
22:4/22:6	1.48 ± 0.15	1.65 ± 0.13	1.25 ± 0.13	1.38 ± 0.30

The comparison between LF and HF groups was carried out by student’s t-test *P < 0.05, **P < 0.01. Differences among three groups HF, DPC and DPS were tested by one-way ANOVA (Turkey’s test) and different letters (a, b, c) indicated different significance at *P* < 0.05

In Table 4, 16:0/22:6, 18:0/18:1, 18:0/20:4, 18:0/22:6, 18:1/20:3, 18:1/22:5 make up the main fatty acid composition for PE molecular species, in which 18:0/20:4 and 18:1/20:3 exhibited significant difference between the four dietary groups. DHA-containing PE, as essential components, could increases membrane fluidity, which played an important role in the composition of membrane phospholipids and brain function. High-fat diet could decrease the level of 18:0/20:4, interestingly, 18:0/20:4 content could be recovered after DHA-PC and DHA-PS intervention. The changes of 18:1/20:3 were in accordance with 18:0/20:4 among the four groups.

**Table 4:** The lipid profile of PE in cerebral cortex after dietary intake of DHA enriched phospholipids

Fatty Acid (%)	LF	HF	DPC	DPS
	Mean ± SD	Mean ± SD	Mean ± SD	Mean ± SD
16:0/18:1	1.48 ± 0.09	1.42 ± 0.14	1.45 ± 0.18	1.48 ± 0.10
16:0/18:2	0.20 ± 0.03	0.13 ± 0.09	0.17 ± 0.05	0.16 ± 0.07
16:0/20:2	0.54 ± 0.08	1.19 ± 0.21 ^**a^	0.33 ± 0.16 ^**b**^	0.45 ± 0.07 ^**b**^
16:0/20:4	1.44 ± 0.40	1.28 ± 0.40	1.27 ± 0.15	1.25 ± 0.07
16:0/22:1	1.50 ± 0.33	1.51 ± 0.42	1.13 ± 0.27	1.24 ± 0.07
16:0/22:4	1.09 ± 0.24	0.35 ± 0.17 ^**b^	0.25 ± 0.10 ^**b**^	0.75 ± 0.06 ^**a**^
16:0/22:5	1.96 ± 0.08	2.75 ± 0.18 ^**a^	1.72 ± 0.39 ^**b**^	1.72 ± 0.12 ^**b**^
**16:0/22:6**	**4.78 ± 1.30**	**5.27 ± 1.26**	**4.54 ± 0.40**	**5.37 ± 1.24**
16:1/20:4	0.10 ± 0.01	0.09 ± 0.01	0.06 ± 0.03	0.07 ± 0.02
16:1/22:6	0.05 ± 0.02	0.03 ± 0.02 ^**ab**^	0.06 ± 0.02 ^**a**^	0.01 ± 0.01 ^**b**^
18:0/16:0	0.12 ± 0.05	0.14 ± 0.09	0.12 ± 0.06	0.11 ± 0.02
**18:0/18:1**	**9.68 ± 2.22**	**9.54 ± 1.58**	**8.72 ± 0.67**	**9.36 ± 0.47**
18:0/18:2	1.91 ± 0.11	2.34 ± 0.98	1.74 ± 0.26	1.75 ± 0.19
18:0/20:1	1.42 ± 0.28	1.51 ± 0.28	1.18 ± 0.21	1.24 ± 0.24
**18:0/20:4**	**9.82 ± 1.88**	**7.13 ± 0.65**^**b**^	**11.08 ± 1.05**^**a**^	**9.31 ± 0.51**^**ab**^
18:0/22:4	1.42 ± 0.35	1.50 ± 0.22	1.13 ± 0.30	1.24 ± 0.11
**18:0/22:6**	**16.05 ± 4.01**	**17.05 ± 3.08**	**16.69 ± 1.29**	**17.27 ± 1.00**
18:1/18:1	3.33 ± 0.27	3.93 ± 1.11	3.14 ± 1.04	3.03 ± 0.59
**18:1/18:2**	**4.69 ± 0.19**	**5.10 ± 0.92**	**5.37 ± 0.31**	**4.53 ± 0.42**
**18:1/20:3**	**9.87 ± 0.56**	7.86 ± 0.21^*b^	**8.87 ± 0.88**^**ab**^	**10.10 ± 1.06**^**a**^
18:1/20:4	1.51 ± 0.11	1.67 ± 0.20	1.46 ± 0.22	1.34 ± 0.23
**18:1/20:5**	**4.57 ± 0.26**	**4.33 ± 1.13**	**5.19 ± 0.49**	**4.37 ± 0.55**
18:1/22:4	0.02 ± 0.01	0.03 ± 0.01	0.03 ± 0.02	0.03 ± 0.01
**18:1/22:5**	**15.08 ± 1.39**	**16.51 ± 1.13**	**15.90 ± 1.25**	**16.32 ± 0.99**
18:1/22:6	1.51 ± 0.09	1.43 ± 0.12	1.51 ± 0.19	1.37 ± 0.13
**18:2/20:4**	**4.57 ± 0.47**	**4.29 ± 0.69**	**5.19 ± 0.77**	**4.33 ± 0.63**
18:2/22:6	0.48 ± 0.06	0.64 ± 0.19	0.71 ± 0.25	0.74 ± 0.29
20:4/20:4	0.50 ± 0.13	0.64 ± 0.19	0.71 ± 0.11	0.74 ± 0.26
20:4/22:6	0.08 ± 0.02	0.08 ± 0.02	0.08 ± 0.01	0.07 ± 0.02
22:4/22:6	0.15 ± 0.02	0.20 ± 0.03 ^*a^	0.12 ± 0.02 ^**b**^	0.15 ± 0.02 ^**b**^
22:6/22:6	0.07 ± 0.02	0.09 ± 0.03	0.10 ± 0.02	0.10 ± 0.04

The comparison between LF and HF groups was carried out by student’s t-test *P < 0.05, **P < 0.01. Differences among two groups HF, DPC and DPS were tested by one-way ANOVA (Turkey’s test) and different letters (a, b) indicated different significance at *P* < 0.05

As the major component of glycerophospholipids, PC is one of the important bioactive substances. It has been reported that there existed close link between PC and disorders including cardiovascular diseases, liver diseases and nervous system diseases [22]. The predominant molecular species of PC was 16:0/18:1. In the cerebral cortex of dementia mice, different dietary conditions did not change the compositional characteristic of PC even if polyunsaturated fatty acids were ingested (Table 5**)**. The PC with low unsaturation could endow the stability of cell membranes.

**Table 5:** The lipid profile of PC in cerebral cortex after dietary intake of DHA enriched phospholipids

Fatty Acid (%)	LF	HF	DPC	DPS
	Mean ± SD	Mean ± SD	Mean ± SD	Mean ± SD
13:0/22:5	0.21 ± 0.03	0.38 ± 0.08 ^**a^	0.10 ± 0.05 ^**b**^	0.14 ± 0.06 ^**b**^
13:0/22:6	1.45 ± 0.15	1.38 ± 0.08	1.52 ± 0.29	1.32 ± 0.18
15:0/18:1	0.35 ± 0.04	0.40 ± 0.07	0.33 ± 0.09	0.32 ± 0.14
**15:0/22:6**	**4.93 ± 0.89**	**5.31 ± 1.03**	**4.67 ± 0.59**	**4.98 ± 0.95**
15:1/20:4	0.21 ± 0.12	0.38 ± 0.05 ^*a^	0.10 ± 0.02 ^**b**^	0.14 ± 0.06 ^**b**^
16:0/14:0	0.26 ± 0.06	0.29 ± 0.08	0.24 ± 0.15	0.25 ± 0.03
16:0/15:0	0.27 ± 0.14	0.26 ± 0.03	0.25 ± 0.13	0.25 ± 0.06
**16:0/16:0**	**14.72 ± 0.77**	**14.05 ± 1.77**	**14.54 ± 1.15**	**13.74 ± 1.88**
16:0/16:1	2.98 ± 0.25	2.99 ± 0.41^**a**^	2.19 ± 0.15 ^**b**^	2.81 ± 0.40 ^**ab**^
16:0/17:0	0.18 ± 0.05	0.20 ± 0.09	0.18 ± 0.03	0.17 ± 0.07
16:0/17:1	0.61 ± 0.11	0.66 ± 0.28	0.56 ± 0.29	0.55 ± 0.16
**16:0/18:1**	**18.34 ± 2.80**	**18.08 ± 2.90**	**19.36 ± 0.58**	**19.27 ± 1.72**
16:0/18:2	1.46 ± 0.32	1.75 ± 0.26	1.69 ± 0.32	1.90 ± 0.05
16:0/19:1	0.23 ± 0.06	0.26 ± 0.03	0.25 ± 0.07	0.23 ± 0.09
16:0/20:3	0.33 ± 0.20	0.31 ± 0.10	0.37 ± 0.21	0.33 ± 0.13
16:0/20:4	5.26 ± 0.70	5.73 ± 0.65	4.44 ± 0.44	5.22 ± 0.76
16:0/22:1	0.18 ± 0.08	0.18 ± 0.05	0.17 ± 0.04	0.17 ± 0.04
**16:0/22:6**	**5.58 ± 0.83**	**5.20 ± 1.14**	**5.92 ± 1.56**	**5.23 ± 0.84**
16:0/24:2	0.04 ± 0.03	0.04 ± 0.02	0.04 ± 0.03	0.04 ± 0.01
16:1/18:2	0.04 ± 0.01	0.04 ± 0.01	0.03 ± 0.02	0.04 ± 0.02
16:1/19:1	0.02 ± 0.01	0.03 ± 0.02	0.02 ± 0.02	0.02 ± 0.01
16:1/20:4	0.13 ± 0.05	0.02 ± 0.01 ^*^	0.02 ± 0.01	0.03 ± 0.02
16:1/22:6	0.15 ± 0.08	0.12 ± 0.04	0.15 ± 0.07	0.14 ± 0.03
16:1/24:7	0.11 ± 0.07	0.13 ± 0.02	0.12 ± 0.03	0.13 ± 0.02
17:0/20:4	0.08 ± 0.07	0.05 ± 0.04	0.07 ± 0.03	0.05 ± 0.02
17:0/24:2	0.01 ± 0.00	0.01 ± 0.01	0.01 ± 0.00	0.01 ± 0.01
17:1/18:1	0.02 ± 0.02	0.03 ± 0.02	0.02 ± 0.01	0.02 ± 0.03
17:1/20:4	0.34 ± 0.09	0.17 ± 0.04 ^*^	0.19 ± 0.04	0.13 ± 0.07
18:0/13:0	0.04 ± 0.03	0.04 ± 0.01	0.04 ± 0.01	0.03 ± 0.01
18:0/16:0	3.74 ± 0.26	3.88 ± 0.85	3.46 ± 0.77	3.70 ± 0.53
18:0/17:1	0.01 ± 0.01	0.02 ± 0.01	0.02 ± 0.01	0.02 ± 0.01
**18:0/18:1**	**6.14 ± 1.23**	**6.01 ± 0.86**	**5.12 ± 0.62**	**6.10 ± 1.45**
18:0/18:2	2.68 ± 0.72	1.96 ± 0.22 ^**b**^	2.97 ± 0.70 ^**ab**^	3.37 ± 0.13 ^**a**^
18:0/20:4	1.10 ± 0.10	0.45 ± 0.08 ^**b**^	3.18 ± 0.84 ^**a**^	0.95 ± 0.20 ^**b**^
18:0/22:1	0.03 ± 0.02	0.04 ± 0.03	0.03 ± 0.02	0.04 ± 0.02
18:0/22:6	3.53 ± 1.04	3.26 ± 0.32	3.66 ± 0.83	3.30 ± 0.88
18:0/24:1	0.02 ± 0.01	0.02 ± 0.01	0.02 ± 0.01	0.02 ± 0.01
18:0/24:2	0.02 ± 0.01	0.02 ± 0.01	0.02 ± 0.01	0.02 ± 0.01
18:1/18:2	0.17 ± 0.04	0.20 ± 0.11	0.23 ± 0.19	0.22 ± 0.17
18:1/18:3	0.90 ± 0.07	1.09 ± 0.21	0.90 ± 0.15	1.22 ± 0.19
18:1/20:4	1.60 ± 0.14	1.78 ± 0.63	1.35 ± 0.48	1.62 ± 0.31
18:1/22:6	1.28 ± 0.35	1.27 ± 0.23	1.48 ± 0.30	1.38 ± 0.29
19:0/22:1	0.01 ± 0.01	0.01 ± 0.02	0.01 ± 0.00	0.01 ± 0.00
20:0/16:0	0.06 ± 0.03	0.06 ± 0.03	0.06 ± 0.04	0.06 ± 0.04
20:0/18:2	0.23 ± 0.10	0.20 ± 0.15	0.21 ± 0.02	0.20 ± 0.05
20:0/20:4	0.28 ± 0.06	0.27 ± 0.14	0.20 ± 0.20	0.22 ± 0.03
20:0/22:4	0.02 ± 0.01	0.02 ± 0.01	0.01 ± 0.01	0.02 ± 0.01
**20:1/14:0**	**19.13 ± 1.23**	**20.36 ± 2.10**	**19.01 ± 3.06**	**19.40 ± 0.71**
20:1/15:0	0.02 ± 0.01	0.02 ± 0.01	0.02 ± 0.01	0.02 ± 0.01
20:1/20:4	0.07 ± 0.03	0.15 ± 0.04 ^**a^	0.03 ± 0.02 ^**b**^	0.04 ± 0.01 ^**b**^
20:1/22:6	0.08 ± 0.01	0.07 ± 0.04	0.07 ± 0.03	0.08 ± 0.03
20:4/22:6	0.26 ± 0.05	0.26 ± 0.05	0.25 ± 0.14	0.25 ± 0.09
22:4/22:6	0.02 ± 0.02	0.03 ± 0.02	0.02 ± 0.02	0.00 ± 0.00
22:5/22:6	0.01 ± 0.00	0.01 ± 0.01^**a**^	0.00 ± 0.00^**b**^	0.00 ± 0.00^**b**^
22:6/22:6	0.06 ± 0.03	0.05 ± 0.01	0.08 ± 0.04	0.06 ± 0.03

The comparison between LF and HF groups was carried out by student’s t-test *P < 0.05, **P < 0.01. Differences among two groups HF, DPC and DPS were tested by one-way ANOVA (Turkey’s test) and different letters (a, b) indicated different significance at *P* < 0.05

Ether-linked phospholipid and plasmalogen have more potent biological activities in brain function compared with glycerophospholipid, however the possible underlying mechanism has not been illustrated yet. It has been reported that the levels of plasmalogen, especially those with polyunsaturated fatty acids, could significantly be decreased in the brain of Alzheimer’s patients [23]. As shown in Fig. 1, the levels of ethanolamine plasmalogen (pPE) and choline plasmalogen (pPC), as the main components of plasmalogen in the brain, did not significantly change. Notably, distinct alteration appeared in the abundance of PUFA-containing plasmalogen. Administration of high-fat diet slightly decreased14:0p/20:4 pPC in comparison with LF group, and DHA-PC instead of DHA-PS obviously recovered its level (Table 6**)**. In the molecular species of pPE, 16:0p/22:6 and 18:0p/22:6 exhibited distinct alteration, which was consistent with DHA-containing PS (Table 7**)**. The level of ether-linked phospholipids, including alkyl ether analog of phosphatidylcholine (ePC) and alkyl ether analog of phosphatidylethanolamine (ePE), was relatively few in glycerophospholipids and did not significantly change among the four diet-treated groups (Table 8 and Table 9).

**Table 6:** The lipid profile of pPC in cerebral cortex after dietary intake of DHA enriched phospholipids

Fatty Acid (%)	LF	HF	DPC	DPS
	Mean ± SD	Mean ± SD	Mean ± SD	Mean ± SD
14:0p/20:4	13.28 ± 3.20	11.22 ± 2.42 ^**b**^	17.00 ± 1.82 ^**a**^	13.03 ± 2.38 ^**ab**^
14:0p/22:6	7.22 ± 1.56	7.71 ± 1.82	8.08 ± 2.36	7.90 ± 1.55
16:0p/20:4	9.48 ± 0.73	9.25 ± 2.16 ^**ab**^	7.12 ± 0.96 ^**b**^	9.96 ± 1.02 ^**a**^
16:1p/21:2	5.22 ± 2.99	4.70 ± 1.34	3.86 ± 0.84	5.92 ± 1.59
18:0p/19:1	4.93 ± 0.72	5.60 ± 1.54	3.90 ± 0.10	4.42 ± 0.54
18:2p/19:1	5.22 ± 0.75	1.94 ± 0.96 ^*b^	3.86 ± 1.12 ^**ab**^	5.92 ± 1.90 ^**a**^
20:0p/19:1	54.65 ± 5.01	59.59 ± 3.60	56.18 ± 3.93	52.85 ± 6.87

The comparison between LF and HF groups was carried out by student’s t-test *P < 0.05. Differences among two groups HF, DPC and DPS were tested by one-way ANOVA (Turkey’s test) and different letters (a, b) indicated different significance at *P* < 0.05

**Table 7:** The lipid profile of pPE in cerebral cortex after dietary intake of DHA enriched phospholipids

Fatty Acid (%)	LF	HF	DPC	DPS
	Mean ± SD	Mean ± SD	Mean ± SD	Mean ± SD
16:0p/16:0	0.12 ± 0.02	0.12 ± 0.05	0.09 ± 0.03	0.10 ± 0.02
16:0p/18:1	2.09 ± 0.30	2.23 ± 0.33	1.95 ± 0.17	2.10 ± 0.19
16:0p/20:4	2.39 ± 0.73	2.15 ± 0.38	1.73 ± 0.12	1.95 ± 0.13
16:0p/22:4	2.40 ± 0.47	2.04 ± 0.19	1.49 ± 0.22	1.84 ± 0.12
16:0p/22:5	0.84 ± 0.25	1.30 ± 0.25 ^*a^	0.12 ± 0.04 ^**b**^	0.16 ± 0.06 ^**b**^
**16:0p/22:6**	**11.58 ± 1.22**	8.55 ± 0.60^*b^	**10.93 ± 1.16**^**a**^	**10.77 ± 1.06**^**a**^
16:0p/24:7	0.38 ± 0.21	0.38 ± 0.06	0.44 ± 0.07	0.39 ± 0.05
18:0p/16:0	0.33 ± 0.08	0.30 ± 0.08	0.24 ± 0.07	0.24 ± 0.06
18:0p/18:1	2.38 ± 0.26	2.41 ± 0.22 ^**ab**^	2.13 ± 0.36 ^**b**^	2.69 ± 0.17 ^**a**^
18:0p/20:1	0.58 ± 0.15	0.56 ± 0.18	0.50 ± 0.18	0.60 ± 0.19
**18:0p/20:3**	**11.56 ± 3.09**	**14.76 ± 2.38**	**14.84 ± 1.74**	**14.16 ± 1.48**
18:0p/20:4	3.25 ± 0.86	3.57 ± 0.65	2.81 ± 0.32	3.10 ± 0.37
18:0p/22:4	4.12 ± 0.95	3.58 ± 0.64	2.82 ± 0.34	3.36 ± 0.26
18:0p/22:5	0.27 ± 0.07	0.37 ± 0.16 ^**a**^	0.11 ± 0.04 ^**b**^	0.15 ± 0.03 ^**b**^
**18:0p/22:6**	**15.94 ± 0.93**	13.88 ± 0.62^*b^	**15.84 ± 0.75**^**a**^	**15.49 ± 0.66**^**a**^
18:1p/16:0	1.96 ± 0.27	2.11 ± 0.40	1.84 ± 0.18	1.99 ± 0.24
18:1p/18:1	4.44 ± 1.23	5.52 ± 0.61	4.19 ± 0.34	4.76 ± 0.62
18:1p/20:1	1.13 ± 0.07	1.01 ± 0.18	0.94 ± 0.21	1.20 ± 0.16
18:1p/20:4	4.03 ± 0.79	3.89 ± 0.34	3.19 ± 0.94	3.82 ± 0.32
**18:1p/20:5**	**10.59 ± 2.17**	**9.55 ± 1.09**	**10.56 ± 1.68**	**9.81 ± 0.38**
18:1p/22:1	0.14 ± 0.04	0.15 ± 0.04	0.13 ± 0.04	0.14 ± 0.03
18:1p/22:4	2.36 ± 0.45	2.21 ± 0.10	1.99 ± 0.46	2.25 ± 0.49
**18:1p/22:5**	**11.55 ± 1.93**	**13.56 ± 1.67**	**14.71 ± 1.45**	**13.08 ± 2.57**
**18:1p/22:6**	**5.53 ± 0.59**	**5.62 ± 0.68**	**6.36 ± 1.21**	**5.77 ± 1.09**
18:2p/20:4	0.00 ± 0.00	0.12 ± 0.02 ^**a^	0.00 ± 0.00 ^**b**^	0.08 ± 0.03 ^**a**^
18:2p/22:6	0.04 ± 0.01	0.06 ± 0.02 ^**a**^	0.05 ± 0.02 ^**a**^	0.01 ± 0.00 ^**b**^

The comparison between LF and HF groups was carried out by student’s t-test *P < 0.05, **P < 0.01. Differences among two groups HF, DPC and DPS were tested by one-way ANOVA (Turkey’s test) and different letters (a, b) indicated different significance at *P* < 0.05

**Table 8:** The lipid profile of ePC in cerebral cortex after dietary intake of DHA enriched phospholipids

Fatty Acid (%)	LF	HF	DPC	DPS
	Mean ± SD	Mean ± SD	Mean ± SD	Mean ± SD
**16:0e/16:0**	**16.18 ± 1.94**	**15.10 ± 1.85**	**17.05 ± 1.51**	**14.57 ± 2.52**
**16:0e/16:1**	**12.74 ± 2.77**	**11.48 ± 3.00**	**11.20 ± 2.12**	**11.40 ± 1.45**
**16:0e/18:1**	**32.72 ± 4.27**	**30.81 ± 4.53**	**34.32 ± 3.85**	**32.86 ± 4.60**
16:0e/18:2	4.88 ± 2.15	4.48 ± 1.24	3.96 ± 1.89	4.52 ± 0.34
16:0e/19:1	0.75 ± 0.54	0.76 ± 0.22	0.40 ± 0.10	0.47 ± 0.09
**16:0e/22:1**	**8.46 ± 1.81**	**11.21 ± 1.11**^**a**^	**8.21 ± 1.43**^**b**^	**8.66 ± 0.93**^**ab**^
**18:0e/16:0**	**7.01 ± 2.35**	**6.13 ± 1.20**	**5.97 ± 1.51**	**5.62 ± 0.58**
18:0e/18:1	1.87 ± 0.88	1.86 ± 0.20	1.87 ± 0.18	2.02 ± 1.46
18:0e/18:2	2.40 ± 0.33	2.56 ± 0.42	2.32 ± 0.36	2.90 ± 0.50
18:0e/22:1	0.88 ± 0.23	1.17 ± 0.45	0.81 ± 0.12	1.00 ± 0.12
**18:0e/22:6**	**7.21 ± 1.37**	**8.56 ± 1.44**	**8.83 ± 1.40**	**10.46 ± 1.68**
18:0e/23:2	0.72 ± 0.26	0.94 ± 0.19	1.12 ± 0.08	1.07 ± 0.50
20:0e/19:1	4.18 ± 0.49	4.94 ± 1.50	3.94 ± 0.50	4.45 ± 0.83

Differences among two groups HF, DPC and DPS were tested by one-way ANOVA (Turkey’s test) and different letters (a, b) indicated different significance at *P* < 0.05

**Table 9:** The lipid profile of ePE in cerebral cortex after dietary intake of DHA enriched phospholipids

Fatty Acid (%)	LF	HF	DPC	DPS
	Mean ± SD	Mean ± SD	Mean ± SD	Mean ± SD
**16:0e/22:4**	**44.68 ± 4.51**	**49.28 ± 3.88**	**49.34 ± 3.41**	**49.09 ± 4.45**
18:0e/18:2	0.66 ± 0.30	0.50 ± 0.12	0.50 ± 0.12	0.73 ± 0.21
**18:0e/20:4**	**53.32 ± 4.55**	**49.28 ± 3.99**	**49.35 ± 3.34**	**49.09 ± 4.46**
18:0e/22:5	1.34 ± 0.32	0.94 ± 0.14	0.81 ± 0.11	1.09 ± 0.31

### PUFA content in glycerophospholipid

The main long chain fatty acids in glycerophospholipid from cerebral cortex are arachidonic acid (AA), eicosapentaenoic acid (EPA), docosapentaenoic acid (DPA) and DHA, which are consistent with those of the whole brain [24]. As the main PUFA of the membrane phospholipid, AA and its metabolites possess the powerful physiological activity in nerve and immune system [25]. AA has higher content in molecular species PE, PC, pPC, pPE and ePE compared with other glycerophospholipids including PS and ePC (Fig. 2). Although there was no significant difference in the level of AA in most glycerophospholipids, such as PC, pPC, pPE and Epe, after the intervention, interestingly, high-fat diet intervention could significantly decrease the level of AA-containing PE, and both DHA enriched phospholipids groups could recover it to that of LF group (Fig. 2b). As presented in Fig. 2, DPA was mainly concentrated in PS, PE and pPE, only the DPA level in PS was different among four different dietary groups. HF and DPS groups could remarkably increase the level of DPA-containing PS, while there was no significance change between DHA-PC group and LF group.

EPA, an important source of n-3 PUFA for the whole body, can effectively prevent cardiovascular diseases and supply essential nutrients for the brain [20]. In the cerebral cortex, EPA was only detected in PE and pPE molecular species, which might be attributed to that PE and pPE were responsible for the membrane fluidity due to the presence of large amounts of polyunsaturated fatty acids [26] (Fig. 2b and e). However, there were no significant differences among these four diet-treated group, indicating that the EPA level in the brain might be relatively stable.

As the most abundant long chain PUFA, DHA exhibited the high levels in the molecular species of glycerophospholipid except for ePE, meanwhile obvious changes were reflected in pPE and PS. In pPE, the relative content of DHA-containing pPE significantly decreased after dietary intake of high-fat diet, which was consistent with the previously reported results that high-fat could accelerate aging and dementia [27]. Notably, the supplementation of DHA enriched phospholipids, including DHA-PC and DHA-PS, obviously increased DHA-containing pPE and PS levels compared with HF group, which reached to that of LF group.

## Discussion

Brain function depends on the homeostasis of nerve system, which was associated with the phospholipid of cellular membrane. The cerebral cortex possesses the most DHA content in the total fatty acid composition compared with hippocampus and other brain regions. More importantly, DHA, especially in the form of plasmalogen, in the cortex decreased the most during the pathological process of AD [28]. Therefore, the changes of lipid profile might be observed in the cerebral cortex under the condition of neurodegenerative disorder after different dietary patterns. In the present study, the main glycerophospholipid molecular species were not significantly changed after administration with DHA enriched phospholipids and high-fat diet for 2 months in SAMP8 mice. The reason might be that the dose of DHA enriched phospholipids was not enough to dramatically alter fatty acid composition in the brain. In our previous study, the intervention of DHA enriched phospholipids at 300 mg/kg body weight could significantly alleviate pathology symptoms in Aβ-induced AD, but not change the whole brain DHA level [11]. The brain is a closed system isolated by blood-brain barrier, and supplementation of exogenous lipids may not cause significant changes in the lipid composition of the brain.

The different cross-regionally peroxidability index are due to changes in the type of unsaturated fatty acid that participates in membrane composition. Owing to the lipid and fatty acid composition, cerebral cortex has high peroxidability index. Therefore, the PUFA content in glycerophospholipid could reflect the oxidative stress status in the brain to some extent. There were significant changes in the levels of PUFA in some specific molecular species, including DPA-containing PS, DHA-containing PS/pPE, and AA-containing PE, among the four dietary treated groups.

Plasmalogens are critical for human health and have established roles in neuronal development, the immune response and as endogenous antioxidants [29]. Plasmalogen was decreased significantly in human brain with AD, in which ethanolamine plasmalogen was the main component [30, 31]. The ethanolamine plasmalogen has good antioxidant activity owing to its vinyl ether double bond and n-3 polyunsaturated fatty acids. Although the overall level of ethanolamine plasmalogen were not observed, the level of DHA-containing pPE were significantly changed after administration with different diets.

It has been reported that both DHA-PC and DHA-PS administration could remarkably mitigate cognitive deficiency in Aβ-induced rats, in which the effect of DHA-PS was better than that of DHA-PC [11]. Moreover, Zhou et al. [32] reported that both DHA-PC and DHA-PS significantly improved the cognitive deficits in SAMP8 mice fed with high-fat diet using maze tests. DHA-PS presented more notable benefits than DHA-PC on inhibiting Aβ pathology, mitochondrial damage, neuroinflammation. In the present study, no remarkable changes were observed in the lipid profile of cerebral cortex in SAMP8 mice after administration with DHA-PC and DHA-PS for 2 months, suggesting us that the alteration of brain lipid composition might not be the main reason for their different efficiencies. The possible underlying molecular mechanism might be closely related to the different polar groups in phospholipids. Cholinergic system played an important role in the treatment for dementia and mental deterioration, and dietary intake of phosphatidylcholine could accelerate the biosynthesis of acetylcholine in vivo [33]. It has been reported that administration of egg PC could improve memory and maze-learning ability in mice with dementia by raising the acetylcholine level in brain [34]. Phosphatidylserine, as an important nutrient for cell membrane, was essential for the activation of several key signaling pathways in neuronal signal transduction. PS supplementation could improve the memory functions of the elderly with memory complaints [35], increase acetylcholine release and the activity of Na_1_, K_1_-ATPase in synaptogenesis [36]. DHA-PC could ameliorate cognitive impairment mainly through cholinergic system, while signal transduction molecules might be the main reason for the effect of DHA-PS on improving memory function.

## Conclusion

Administration with DHA-PC and DHA-PS was reported to improve brain function, and there was a close association between lipid homeostasis and human health. In the present study, the lipid profile in cerebral cortex was determined by lipidomics after administration with DHA-PC and DHA-PS for 2 months in high-fat diet induced SAMP 8 mice with cognitive deficiency. Results showed that high-fat diet could significantly decrease the levels of DHA-containing PS/pPE, DPA-containing PS, and AA-containing PE. Interestingly, DHA-PC and DHA-PS remarkably recovered the lipid homeostasis compared with that of SAMP 8 mice feeding low-fat diet although the main glycerophospholipid molecular species were not significantly changed. It is necessary to find more lipid biomarkers for the prevention and diagnosis of AD. These results might provide a potential novel therapy strategy and direction of dietary intervention for patients with cognitive decline.

## Data Availability

All the data generated or analyzed during this study are included in this published article.

## Abbreviations

AA: Arachidonic acid
Aβ: Amyloid beta
AD: Alzheimer’s disease
Akt: Serine/threonine kinase
DHA: Docosahexaenoic acid
DHA-PC: DHA enriched phosphatidylcholine
DHA-PS: DHA enriched phosphatidylserine
DPA: Docosapentaenoic acid
EPA: Eicosapentaenoic acid
ePC: Alkyl ether analog of phosphatidylcholine
ePE: Alkyl ether analog of phosphatidylethanolamine
HPLC-ELSD: High-performance liquid chromatography with evaporative light scattering detector
IL-1β: Interleukin 1 beta
LPE: Lyso-phosphatidylethanolamine
LPC: Lyso-phosphatidylcholine
PC: Phosphatidylcholine
PE: Phosphatidylethanolamine
PPAR-α: Peroxisome proliferator-activated receptors
pPC: choline plasmalogen
pPE: Ethanolamine plasmalogen
PS: Phosphatidylserine
PUFA: Polyunsaturated fatty acid
Raf: Root abundant factor
SAMP8 mice: Senescence accelerated mouse-prone 8
SPF: Specific pathogen free
TNF-α: Tumor necrosis factor alpha
